# National trends of malpractice-related cardiovascular mortality within the United States, 1999–2020

**DOI:** 10.1097/MS9.0000000000002399

**Published:** 2024-07-23

**Authors:** Hamza Ashraf, Haider Ashfaq, Neha Saleem Paryani, Shanza Malik, Ali Tariq Shaikh, Aalaa Saleh, Jehanzeb Ahmed Khan, Ishaque Hameed

**Affiliations:** Departments ofa Cardiology; b Medicine, Allama Iqbal Medical College, Lahore; c Department of Medicine, Dow University of Health Sciences, Karachi, Pakistan; d Department of Medicine, United Health Services (Wilson Medical Center), Wilson, NC; e Department of Cardiovascular Medicine, Oklahoma University, Norman, OK, USA; f Faculty of Medicine, Lebanese University, Beirut, Lebanon; g Department of Medicine, Medstar Health Baltimore, Baltimore, Maryland

**Keywords:** cardiovascular disease, CDC WONDER, geriatrics, malpractice, mortality, USA

## Abstract

Cardiovascular disease (CVD) stands as the leading cause of mortality in the USA, claiming a life every 33 seconds, while cardiology ranks among the top three specialties with malpractice-related claims. The authors’ study aims to scrutinize sex disparities in CVD-related mortality linked with malpractice among the elderly population (≥65 years) in the USA. Data pertaining to malpractice incidents in CVD treatment spanning from 1999 to 2020 were sourced from the CDC Wonder database. Age-adjusted mortality rates (AAMRs) per 1,000,000 individuals were computed. Joinpoint regression analysis was used to determine the annual percent changes (APCs) with a 95% CI, stratified across variables such as age, race/ethnicity, census region, and urban or rural settings. Over the investigated period, 2432 deaths in the US were attributed to CVD-related malpractice, with an AAMR of 2.7. Initially stable (1999–2004), mortality rates experienced a significant decline until 2020. Females consistently exhibited a higher AAMR (2.7) than males (2.6). Notably, NH Black females recorded the highest AAMR (3.1), while NH Black males and NH Asian females reported the lowest (2.5). Furthermore, NH White males demonstrated a higher AAMR (2.7) than NH Black males (2.5); conversely, NH Black females exhibited a higher AAMR (3.1) than NH White females (2.7). Mortality rates were notably elevated in the West compared to the South, with both urban and rural areas indicating higher AAMRs in females. The authors’ findings underscore the necessity for targeted interventions to address the pronounced disparities, particularly among NH Black women, individuals in the West, males, and urban locales.

## Introduction

Highlights

- Cardiovascular disease is the leading cause of death in the US, with cardiology ranking high in specialties with malpractice claims.
- Malpractice in cardiovascular care accounts for 2.7 deaths per 1 million individuals of age older than 65 in the United States.
- Non-Hispanic Black women, females in the West, males aged older than or equal to 85, and urban areas face higher mortality rates.
- It is crucial to implement targeted interventions in order to tackle the inequalities and offer patients with more equitable and superior quality healthcare.

Cardiovascular disease (CVD), the leading cause of death for both men and women in the United States, claims a life every 33 seconds across most racial and ethnic groups^1,2^. Malpractice incidents in cardiology are not uncommon. Jena *et al.* ^3^ highlighted cardiology among the top three specialties with malpractice-related claims, with a notable percentage of 18.9% in thoracic-cardiovascular surgery. Quinn *et al.* ^4^ also delved into cardiovascular malpractice cases and concluded that cardiovascular cases significantly resulted in death compared to non-cardiovascular cases. The recent surge in malpractice complaints has had a significant effect on patient health outcomes and the mental well-being of physicians. Patients affected by medical errors may experience immediate consequences such as changes in their interactions with healthcare professionals, influenced by their personality, and emotional distress and regression among the patient’s relatives^5^. Furthermore, patients may incur additional medical costs, require further treatment, lose wages, and suffer other damages due to medical negligence^6^. On the other hand, medical errors and adverse events not only affect doctors’ emotional well-being and quality of life but also influence their professional practice and conduct, highlighting the need for healthcare administrations to provide support and counseling services to affected professionals^7^. Understanding the impact of medical errors on mortality rates can lead to improved patient safety measures and quality of care, helping to prevent avoidable deaths^8^. It can also contribute to enhancing healthcare provider accountability, reducing legal disputes, and increasing public trust in healthcare facilities^9,10^. The data regarding the impact of this malpractice on mortality and demographic disparities is very limited. The purpose of this study is to evaluate the trends and gender disparities in CVD-related mortalities due to malpractice in the United States from 1999 to 2020.

## Methods

The data on malpractice in the treatment of CVD has been retrieved from CDC Wonder (Centers for Disease Control and Prevention Wide-Ranging Online Data for Epidemiologic Research) database^11^ for the years 1999–2020. The Multiple Cause-of-Death Public Use records were studied to identify deaths where CVD was listed as the underlying cause of death and malpractice incidents related to medical or surgical care were listed as either underlying or contributing causes of death. CVD deaths were identified using International Classification of Diseases, Tenth Revision, Clinical Modification (ICD-10-CM) codes I00–I99, while codes Y60–Y69 were used to extract data on malpractice-related deaths in patients aged 65 and older. This study adhered to STROBE (Strengthening the Reporting of Observational Studies in Epidemiology) guidelines^12^ and used a de-identified government-issued dataset.

Data on year, population size, location of death, demographics, urban-rural classification, and census region were extracted. Demographics included sex, age, and race/ethnicity, with location of death encompassing medical facilities, home, hospice, and nursing home/long-term care facilities. Race/ethnicity was categorized as non-Hispanic (NH) White, NH Black or African American, Hispanic or Latino, NH American Indian or Alaskan Native, and NH Asian or Pacific Islander. Following the USA Census Bureau 2013 definition, areas with populations of 50 000 or more were designated as urban, whereas areas with populations below 50 000 were designated as rural^13^.

Crude mortality rates were calculated by dividing the number of malpractice-related CVD deaths by the corresponding U.S. population of that year. The age-adjusted mortality rates (AAMRs) were determined by standardizing CVD deaths due to malpractice to the U.S. population of the year 2000^13^. This approach yields a weighted mean, facilitating unbiased comparisons of mortality rates across diverse populations or time periods. In the joinpoint regression analysis conducted using the joinpoint Regression Program 5.0.2^14^, we calculated the annual percentage changes (APCs) and average annual percentage changes (AAPCs) in AAMR, along with 95% CI. This software detects significant shifts in AAMR over time by applying log-linear regression models to periods with temporal variations. APCs for the AAMRs were computed at the identified segments joining the points, utilizing the Monte Carlo permutation test^15^. APCs were deemed to be either increasing or decreasing if the slope indicating the change in mortality was significantly different from zero, confirmed through two-tailed *t*-tests. A *P* value equal to or below 0.05 indicates statistical significance.

## Results

**Figure 1:**
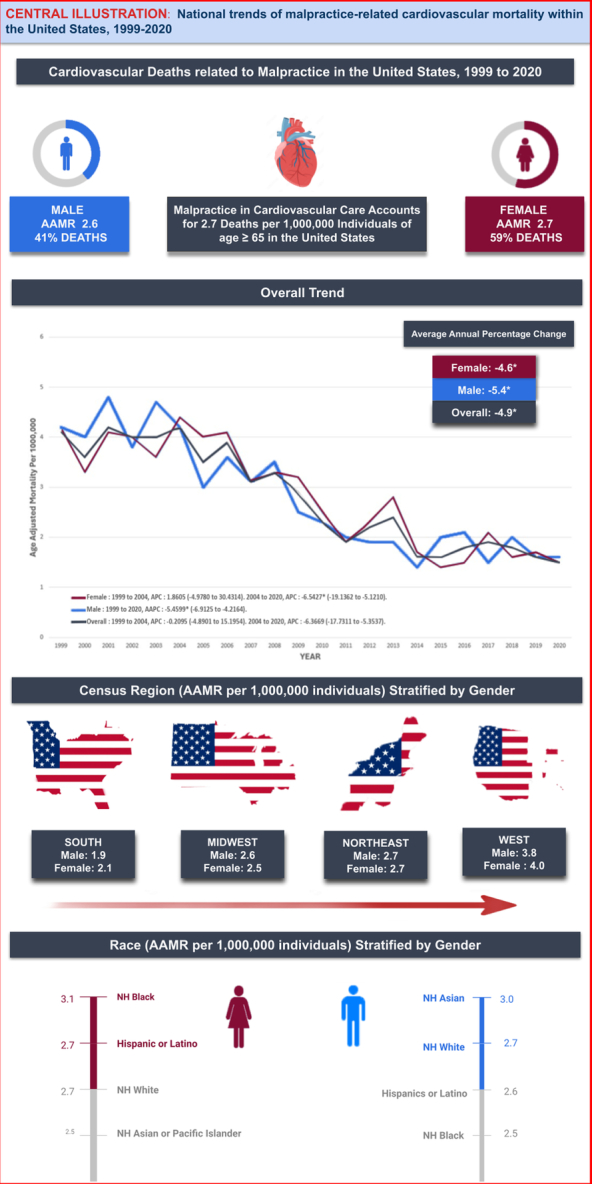
Cardiovascular mortality related to malpractice within the United States. AAMR, Age-adjusted mortality rate; NH, non-Hispanic.

Between 1999 and 2020, a total of 2432 CVD-related deaths occurred due to medical and surgical malpractice in individuals aged older than or equal to 65, among these, 59% were female and 41% were males. 93.5% of the total deaths occurred in medical facilities, with the age-group 75–84 years contributing the majority (46.1%) of the overall deaths. The overall AAMR approximately reduced by 50% from the year 1999–2020, with an AAPC of −4.9* (95% CI: −6.9 to −3.7). Initially there was no change in mortality rate from 1999 to 2004 (APC: −0.21; 95% CI: −4.90 to 15.20), followed by a rapid decline in AAMR till 2020 (APC: −6.37*;95% CI: −17.73 to −5.35). Throughout the study period, females exhibited a consistently higher AAMR (2.7; 95% CI: 2.6–2.9) with an AAPC of −4.61*(95% CI: −6.99 to −2.52) as compared to males (AAMR: 2.6; 95% CI: 2.5–2.8 & AAPC: −5.46*; 95% CI: −6.91 to −4.22) [* indicates that the APC or AAPC is significantly different from zero at the alpha = 0.05 level]. Males of age 85+ years reported significantly higher AAMR (4.8; 95% CI: 4.1–5.4) than females of the same age-group (AAMR 3.7, 95% CI: 3.3–4.1). Among different sex-stratified racial/ethnic groups, NH Black females had the highest overall AAMR (3.1; 95% CI: 2.6–3.6) while NH Black males (2.5; 95% CI: 1.9–3.2) and NH Asian females displayed the lowest overall AAMR (2.5; 95% CI: 1.8–3.3). The AAMR was higher in NH White males (2.7; 95% CI: 2.6–2.8) compared to NH Black males (2.5; 95% CI: 1.9–3.2). Conversely, NH Black females exhibited a higher AAMR (3.1; 95% CI: 2.6–3.6) than NH White females (2.7; 95% CI: 2.6–2.9). When stratified by Census Region, West showed the highest overall AAMR (3.9; 95% CI: 3.6–4.2) followed by Northeast (AAMR: 2.7; 95% CI: 2.4–2.9), Midwest (AAMR: 2.6; 95% CI: 2.3–2.8), and South (AAMR: 2; 95% CI: 1.8–2.1). Males and females in West portrayed two-fold higher mortality rates (AAMR: 3.8; 95% CI: 3.4–4.2 & AAMR: 4; 95% CI: 3.6–4.3, respectively) when compared with males and females in the South region (AAMR: 1.9; 95% CI: 1.7–2.1 and AAMR: 2.1; 95% CI: 1.9–2.3, respectively). The AAMR was higher in Urban areas (2.7; 95% CI: 2.6–2.9) when compared with Rural areas (2.4; 95% CI: 2.2–2.6). In both urban and rural areas, females exhibited higher AAMRs (2.8; 95% CI: 2.6–2.9 and 2.5; 95% CI: 2.1–2.8, respectively), compared to males (AAMR: 2.7; 95% CI: 2.5–2.9 and AAMR: 2.3; 95% CI: 1.6–2.6, respectively. [Fig. 1].

## Discussion

Medical error is still the third biggest cause of death in the United States, following cancer and cardiovascular disease^16^. However, an overall decreasing trend was observed in cardiovascular mortality related to malpractice, with the AAMRs decreasing from 4.1 in 1999 to 2.7 in 2020. This trend was consistent among males as well as females. This decline can be ascribed to multiple factors, including advances in medical practices and health information technology. Health information technology enhances patient safety by reducing medication errors, reducing adverse drug reactions, and improving compliance to practice guidelines^17^. One such initiative was the PROMISES Project that aimed to decrease malpractice and safety risks in outpatient practice by restructuring the management of referrals, medications, and tests and focusing on improving communication between staff and patients^18^.

Among racial trends, NH Blacks showed the highest mortality rates. This can be due to several reasons, including socioeconomic factors, implicit bias, and health literacy, etc. Disparities between Black and White Americans persist in medical treatment and health outcomes. One reason is that physicians sometimes hold implicit racial biases that favor White (over Black) patients^19^. A recent study by Thomas *et al.* ^20^ showed that there were significantly more employee-related claims for White patients as compared to Black Patients. We also observed that females exhibited higher AAMRs than males and this pattern was the most pronounced for NH Black females having an AAMR of 3.1 while their male counterparts had an AAMR of 2.5. This can be explained in part because, after an acute cardiovascular (CV) event, women have a greater death rate and a worse prognosis than males^21^. The REWIND Trial also demonstrated that gender disparities exist in the prescription of cardiovascular treatment drugs such as statins and aspirin between men and women^22^. The existence of disparities even in a trial setting shows the impact of potential inequalities in daily practice as well^22^. Furthermore, certain cardiovascular diseases, such as MI with non-obstructive coronary disease and ischemia with non-obstructive coronary disease are more common in women. Such diseases are associated with a high rate of misdiagnosis^23^. It has also been reported that women face challenges in receiving timely and appropriate care, leading to adverse cardiovascular events post-pregnancy^24^. Similarly, Patel *et al.* ^25^ concluded that women under 55 years of age and NH Black individuals experiencing acute cardiac ischemia symptoms are less likely to be hospitalized compared to their male and white counterparts owing to inherent biases contributing to missed diagnosis. Cognitive-related errors, influenced by subconscious biases, affect how physicians assess patients’ symptoms. Training physicians to recognize and address these biases can help reduce missed diagnoses^25^. It is evident from previous literature that mortality rates can be decreased in women with acute myocardial infarction (AMI) by increased use of percutaneous coronary interventions (PCI)^26^. Furthermore, initiatives such as the American Heart Association’s Go Red for Women Campaign^27^, the Heart Health Centers for Women’s healthcare delivery model proposal^28^, the Women’s Health Initiative^29^ and the Drug Trials Snapshots Program^30^ can help address and reduce gender disparities in cardiovascular care. By further investigating the gender and racial trends, we found out that NH Black females had higher AAMRs than NH White females. In the US, racial healthcare disparities are widely documented, and implicit race bias is one possible cause^31^.

AAMRs were reportedly higher for both males and females living in urban areas as compared to rural areas. This may be attributed to the prevalence of complex CVD procedures primarily performed in urban-based specialized hospitals. Since rural hospitals may not have the ability to perform specialized repairs, they require transfers to well-equipped hospitals causing a delay in specialized care^32^. As medical procedures become more complex, the associated risks increase in a directly proportional manner^33^. Additionally, rural areas often lack properly staffed and equipped medical facilities, resulting in longer procedure times, more bleeding complications, and higher mortality rates for patients undergoing cardiac procedures^34^. Despite improvements in medical technology and the use of advanced techniques in the diagnosis and treatment, the complaint rate of cardiovascular surgeons is increasing and informing the patient and their companions about the side effects of these high-risk surgeries with full disclosure can be effective in the prevention of malpractice and physician mistakes^35^. We also observed that people living in the West region demonstrated twice the AAMR values than those living in the South supported by the fact that the highest percentage of malpractice-related litigation was reported in California^36^.

### Limitations

This study has several limitations. First, as information available on the CDC WONDER database is obtained from death certificates and classified through ICD codes, missed or inaccurate diagnoses can lead to incorrect categorization of CVD or malpractice as causes of mortality^37^. Second, malpractice remains an under-classified condition on death certificates, with the literature revealing severe under-reporting as a cause of mortality. This can lead to an insufficient representation of the number of deaths where malpractice was a contributing factor. Alternatively, increased awareness in recent years or potential improvement in coding could have impacted our results. Third, categories of race/ethnicity can be identified incorrectly, leading to misrepresentation of data^38^.

## Conclusion

In conclusion, our findings highlight a notable decrease in malpractice-related CVD mortality rates over the examined period. However, disparities persist, with NH Blacks, women, urban areas, and the Western region consistently demonstrating higher mortality rates. These disparities underscore the urgent need for targeted interventions to mitigate inequities and enhance the delivery of equitable, high-quality care to all patient populations.

Looking forward, future research endeavors should delve deeper into completely understanding the underlying mechanisms contributing to the disparities among all demographics. Initiatives should be taken to increase the representation of women and minority groups in cardiovascular clinical trials. Prospective studies could investigate the multifaceted interplay of healthcare system factors, socioeconomic determinants, and patient-specific variables in shaping malpractice-related CVD mortality outcomes so we can better inform evidence-based interventions aimed at reducing disparities and improving cardiovascular health outcomes for all individuals.

## Ethical approval

Ethics approval was not required for this review, given that all the data used are publicly accessible from CDC WONDER database and de-identified.

## Consent

Informed consent was not required for this review, given that all the data used are publicly accessible from CDC WONDER database and de-identified.

## Source of funding

Not applicable.

## Author contribution

H.A.: data curation, formal analysis, methodology, writing—original draft, writing—review and editing. H.A.: formal analysis, writing—original draft, writing—review and editing. N.S.P.: investigation, writing—review and editing. S.M.: writing—review and editing. A.T.S.: writing—review and editing. A.S.: writing—review and editing. J.A.K.: writing—review and editing. I.H.: conceptualization, project administration, writing—original draft, writing—review and editing.

## Conflicts of interest disclosure

The authors declare no conflicts of interest.

## Research registration unique identifying number (UIN)

Not applicable.

## Guarantor

Hamza Ashraf, Haider Ashfaq.

## Data availability statement

All data are freely available from the CDC WONDER database.

## Provenance and peer review

Not commissioned, externally peer-reviewed.
